# Keratin Protein-Catalyzed Nitroaldol (Henry) Reaction and Comparison with Other Biopolymers

**DOI:** 10.3390/molecules21091122

**Published:** 2016-08-25

**Authors:** Marleen Häring, Asja Pettignano, Françoise Quignard, Nathalie Tanchoux, David Díaz Díaz

**Affiliations:** 1 Institute of Organic Chemistry, University of Regensburg, Universitätsstr 31, Regensburg 93053, Germany; Marleen.Haering@chemie.uni-regensburg.de (M.H.); asja.pettignano@enscm.fr (A.P.); 2 Institute Charles Gerhardt Montpellier-UMR 5253 CNRS/UM/ENSCM, Matériaux Avancés pour la Catalyse et la Santé, 8 rue de l'École Normale, Cedex 5, Montpellier 34296, France; francoise.quignard@enscm.fr (F.Q.); nathalie.tanchoux@enscm.fr (N.T.); 3 IQAC-CSIC, Jordi Girona 18–26, Barcelona 08034, Spain

**Keywords:** keratin, biopolymer, C-C bond formation, nitroaldol reaction, Henry reaction

## Abstract

Here we describe a preliminary investigation on the ability of natural keratin to catalyze the nitroaldol (Henry) reaction between aldehydes and nitroalkanes. Both aromatic and heteroaromatic aldehydes bearing strong or moderate electron-withdrawing groups were converted into the corresponding β-nitroalcohol products in both DMSO and in water in the presence of tetrabutylammonium bromide (TBAB) as a phase transfer catalyst. Negligible background reactions (i.e., negative control experiment in the absence of keratin protein) were observed in these solvent systems. Aromatic aldehydes bearing electron-donating groups and aliphatic aldehydes showed poor or no conversion, respectively. In general, the reactions in water/TBAB required twice the amount of time than in DMSO to achieve similar conversions. Moreover, comparison of the kinetics of the keratin-mediated nitroaldol (Henry) reaction with other biopolymers revealed slower rates for the former and the possibility of fine-tuning the kinetics by appropriate selection of the biopolymer and solvent.

## 1. Introduction

Keratin is one of the most abundant non-food proteins and represents a group of insoluble, cysteine-rich and stable filament-forming materials derived primarily from wool, hair, feathers, beaks, hooves, horns and nails [1]. Depending on their sulfur content, keratins can be classified as soft or hard keratins. Soft keratins, with a cysteine content up to 2% and found in outer layers of the epidermis and hair core, have a weaker mechanical and chemical stability. Hard keratins with a cysteine content of 10%–14% in hair, wool and skin, exhibit a higher resistance to thermal and chemical influences due to higher content of disulfide bridges [2]. Keratins contain a central ~310 residue domain with four segments in α-helical conformation that are separated by three short linker segments in β-turn conformation [3]. The complete amino acid sequence of keratins obtained from different sources has been reported in several publications [4,5,6,7], making the protein a suitable candidate to be tested as a catalyst. Recently, keratinous materials have attracted increasing attention as an important source of renewable biomaterials, especially since keratin wastes have been estimated to be more than 5 million tons per year [8], being used after degradation for different applications including scaffolds for tissue engineering [9,10] and support matrices for catalytic metal nanoparticles [11], among others [12].

On the other hand, the nitroaldol (Henry) reaction is a highly versatile C-C bond-forming reaction between an aldehyde or ketone and a nitroalkane to yield β-nitroalcohols, which can be easily modified into valuable building blocks [13,14,15,16,17]. As part of one of our research programs devoted to understanding the potential catalytic role of different biopolymers [18,19,20,21], we report here for the first time the catalytic properties of keratin protein towards the nitroaldol (Henry) reaction as well as a critical comparison with the performance of other natural polymers in the same process.

## 2. Results and Discussion

The nitroaldol (Henry) reaction between 4-nitrobenzaldehyde (**1a**, 0.1 mmol) and nitromethane (**2a**, 1.0 mmol) was chosen as a model reaction to demonstrate the potential ability of keratin to promote C-C bond formation under mild conditions. Preliminary experiments using 10 mg of keratin in different solvents showed no formation of the desired β-nitroalcohol **3a** in toluene, THF or CH_3_CN (Table 1, entries 1–3), and very low yields of **3a** (ca. 10%) were obtained in CH_2_Cl_2_, CHCl_3_ or H_2_O (Table 1, entries 4, 5 and 7). However, DMSO and water containing tetrabutylammonium bromide (TBAB) as phase-transfer catalyst were found to be appropriate solvents allowing the selective formation of **3a** in modest yields at room temperature with no or very low background reaction (Table 1, entry 6 and entry 8). Thus, formation of **3a** could be unequivocally ascribed to the intrinsic catalytic activity of the protein. These results are similar to those obtained with other biopolymers [19]. We found that the keratin loading could be reduced to 2 mg without detriment in the product yield (data not shown). However, 10 mg were used in most experiments for practical reasons. In addition, the reaction can be scaled up (i.e., 1 mmol of **1a**) without detriment on the product yield.

With these preliminary results in hand, we examined the substrate scope of the reaction using different aldehydes in combination with nitromethane or nitroethane both in DMSO (Table 2) and H_2_O/TBAB (Table 3) at room temperature. Aromatic aldehydes bearing strong electron-withdrawing substituents were easily converted in DMSO into the corresponding β-nitroalcohols **3a**–**f** in excellent yields regardless the position of the substituent (*ortho*-, *meta*-, *para*-) (Table 2, entries 1–6). Weaker acceptor substituents afforded the corresponding product **3** in moderate yields (Table 2, entries 7, 8, 10, 12, 15). Interestingly, here the *meta*-position (Table 2, entry 9) of the substituent seemed to be more favored, giving rise to **3i** in good yield and twice as much than the *para*-substituted substrate (Table 2, entry 8). In contrast, benzaldehyde or aromatic aldehydes bearing electron-donating substituents yielded only trace amounts of the expected product (Table 2, entries 11, 13,14). Although we did not perform these experiments under longer reaction times and/or higher temperatures, it is expected that these factors could enhance to some extent the yields as we have observed with other biopolymers [19]. Heteroaromatic aldehydes such as 2-pyridinecarboxaldehyde (Table 2, entry 16) lead to the desired product in excellent yield, whereas furfural (Table 2, entry 18) and 1-naphthaldehyde (Table 2, entry 19) proceeded only with low yields.

Regarding the nature of the nucleophile, the use of nitroethane instead of nitromethane provided slightly higher yields of the desired product albeit with no or little diastereoselectivity favoring the *syn* diastereomer (Table 2, entries 2, 6, 17 vs. 1, 5, 16, respectively). Slight *anti*-diastereoselectivy was observed only with 3-nitrobenzaldehyde (Table 2, entry 4). Relative configurations were assigned by comparison with ^1^H-NMR data reported in the literature. For instance, in the model reaction between **1a** and **2b**, the *anti* diastereomer is characterized by a doublet at 4.85 ppm (*J* = 8.3 Hz), whereas the *syn* diastereomer shows the doublet at 5.41 ppm (*J* = 2.4 Hz). These results are similar to those obtained with other biopolymers such as gelatin [19] and suggest that the acidity of the nitroalkane (nitroethane pKa = 8.6; nitromethane pKa = 10.2) constitutes in most cases here a more important factor than steric effects [22]. It is also worth mentioning that aliphatic aldehydes such as isobutyraldehyde were not converted (data not shown). Moreover, despite the α-helical chiral structure of the keratin, HPLC analysis of the reaction mixtures showed no enantioselection, as we have previously observed with other biocatalysts in the same reaction [19,20,21].

Although the desired β-nitroalcohols **3** were also obtained in H_2_O/TBAB, the reactions took two times longer than in DMSO to achieve similar conversions. In H_2_O/TBAB the substituent position in the aldehyde **1** was found to have higher influence than in DMSO. For example, strong electron-withdrawing substituents in *ortho-* and *para-*position favored the reaction (Table 3, entries 1 and 5), whereas a significant decrease of the yield was observed with the same substituent placed in *meta-*position (Table 3, entry 3). The reasons for the differences observed in H_2_O/TBAB vs. DMSO may be related to the solubilization of the reactants and/or involve important variations in the mechanistic pathways, which remain unclear and will be investigated in future research. Furthermore, the expected products were obtained in good yields also with 4-cyanobenzaldehyde (Table 3, entry 12) and 2-pyridine carboxaldehyde (Table 3, entry 17). Other aldehydes were converted less efficiently. Furthermore, the use of nitroethane instead of nitromethane provided significant higher yields of the desired product (Table 3, entries 2, 6, 13, 18 vs. 1, 5, 12, 17, respectively) albeit with almost negligible diastereoselectivity (Table 3, entries 6, 18). It is worth to mention that the replacement of TBAB by other ionic materials such as 1-butyl-3-methylimidazolium hexaflorophosphate (BMIM-PF_6_) afforded the desired compound albeit in lower yield even at longer reaction time (Table 3, entry 1).

**Figure 1:**
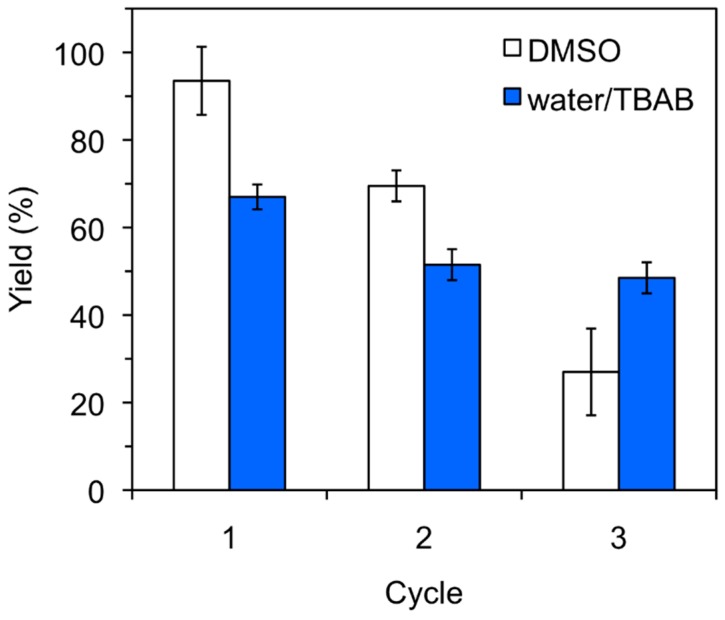
Typical recycling experiments for keratin-catalyzed nitroaldol (Henry) reaction in DMSO and H_2_O/TBAB. Reaction conditions: 4-Nitrobenzaldehyde (**1a**, 15.1 mg, 0.1 mmol), nitromethane (**2a**, 54 μL, 1.0 mmol), solvent (0.5 mL), keratin powder (10 mg), room temperature, 24–48 h. Yields correspond to ^1^H-NMR values obtained from at least two experiments. The error bars represent the standard deviation of the measurements. For the experiments in H_2_O/TBAB, addition of new TBAB after each cycle (i.e., 32.2 mg, 0.1 mmol) was necessary in order to ensure a constant concentration during the reactions as confirmed by NMR analyses of the reaction mixtures.

The heterogeneous nature of the protein catalyst allowed its recovery from the reaction mixture and its reuse for further cycles. However, a gradual deactivation of the catalyst could be observed in both organic and aqueous media, although the reduction of the catalytic activity was clearly more pronounced in DMSO (Figure 1). Such behavior has also been observed with other biopolymers [19,20,21] and could be associated to loss of catalyst loading (e.g., inefficient isolation by filtration) after each cycle and/or formation of intermediate linear or cyclic aminals that could block catalytic sites of the protein. These potential competitive processes are somehow more critical in DMSO than in aqueous solution; however, the reasons behind these differences remain unclear.

**Figure 2:**
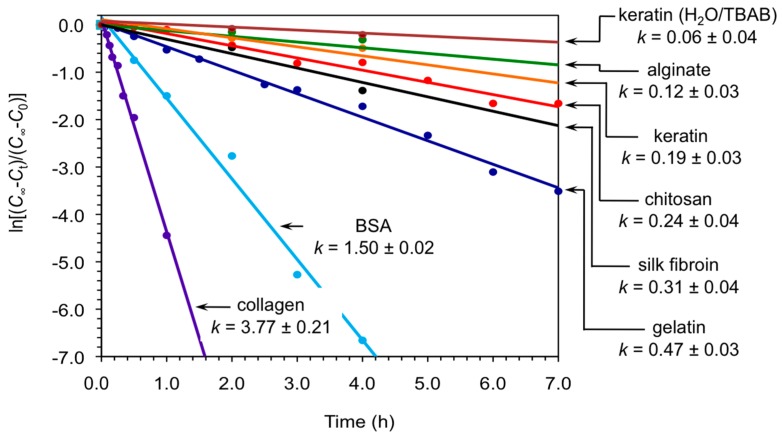
First-order kinetics plots for the nitroaldol (Henry) reaction between **1a** and **2a** catalyzed by different biopolymers in powder form. Unless otherwise indicated, reactions were made in DMSO as described in the text and experimental section. Apparent rate constants are given in units of h^−1^. Each data point represents the average of at least two independent measurements. C_infi_ = final concentration, at infinite time; C_t_ = concentration at given time t; C_0_ = initial concentration at t = zero time.

At this point, we carried out kinetic analyses of the model reaction between **1a** and **2a** catalyzed by keratin in both DMSO and H_2_O/TBAB under the described conditions. The results were compared with the performance of other biopolymers as biocatalysts for the same reaction that we have previously studied in our group and others (i.e., chitosan [18], gelatin [19], collagen [19], bovine serum albumin (BSA) [19,23], silk fibroin [20] and alginate [21]) under optimized conditions. Figure 2 shows the first-order kinetics corresponding to each biopolymer within the first hours of reaction. In general, powdered keratin displayed rate constants in the range of aerogel calcium alginate [21] and significantly below the other biopolymers demonstrating the possibility of fine-tuning the kinetics of the nitroaldol (Henry) reaction by appropriate selection of the biopolymer and solvent system. It is worth mentioning that although these biopolymers are not superior to standard base catalysts such as tetramethylethylenediamine [16], the former avoided the formation of byproducts and allowed to work under ecofriendly and heterogeneous conditions. However, far beyond the interest of these materials as standard catalyst, their mechanism of action to promote C-C bond formation reactions may be more relevant within the context of biological evolution.

## 3. Materials and Methods

### 3.1. Materials

Unless otherwise indicated, analytical grade solvents and reactants were commercially available and used as received without further purification. Deionized water was used for experiments in aqueous solutions. Aldehydes (purity by GC > 98%) were purchased from TCI Europe (TCI Europe, Zwijndrecht, Belgium). Keratin extracted from wool (CAS 69430-36-0, Cat. Nr. AB 250197) was purchased from ABCR. Tetrabutylammonium bromide (CAS 1643-19-2, Cat Nr. 86860) was purchased from Fluka (Fluka Chemical Corp., Milwaukee, WI, USA). 1-Buthyl-3-methylimidazolium hexafluorophosphate (BMIM-PF_6_) was purchased from TCI Europe (CAS 174501-64-5, Cat. Nr. B2320).

### 3.2. Methods

^1^H-NMR spectra were recorded on Avance 300 or Avance 400 spectrometers (Bruker, Billerica, MA, USA) at 25 °C. Chemical shifts for ^1^H-NMR were reported as δ, parts per million, relative to external standards. Yields were determined by ^1^H-NMR analyses of the crude product in CDCl_3_ using dimethyl acetamide (0.1 mmol, 9.2 µL) as internal standard after complete work-up of the reaction. Enantiomeric excess was evaluated by chiral HPLC (Agilent Technologies, Santa Clara, CA, USA) (column: Phenomenex (Phenomenex, Aschaffenburg, Germany) Lux Cellulose-1, 4.6 mm × 250 mm, 5 µm, eluents: *n*-heptane, *i*-propanol 70:30, flow: 0.5 mL/min). Relative configurations were assigned by comparison with ^1^H-NMR data reported in the literature. For example, in the model reaction between **1a** and **2b**, the *anti* diastereomer was identified by a doublet at 4.85 ppm (*J* = 8.3 Hz), whereas the *syn* diastereomer showed the doublet at 5.41 ppm (*J* = 2.4 Hz). For kinetics calculations, the ^1^H-NMR analyses of the reaction mixtures were performed in the presence of an internal standard as above indicated. In general, given yield values correspond to the average of at least two independent measurements with STDV ± 2%–4%. Among various kinetics models, the straight lines shown in the kinetics plots correspond to the best fit of the first-order model (e.g., (nitromethane) ≥ (aldehyde)).

### 3.3. General Procedure for Keratin-Catalyzed Nitroaldol (Henry) Reaction

Keratin (10 mg) was added in one portion to a mixture of 4-nitrobenzaldehyde (**1a**, 0.1 mmol, 15.1 mg), nitromethane (**2a**, 1.0 mmol, 54 μL) and solvent (0.5 mL) placed into a 4-mL screw-capped vial. The resulting reaction mixture was gently shaked in an orbital shaker (150 rpm) for the appropriate time at room temperature. After completion, water (1 mL) was added. The reaction mixture was extracted with EtOAc (4 × 1.5 mL), dried over anhydrous sodium sulfate, filtrated and evaporated under reduced pressure. Yield was determined by ^1^H-NMR of the crude product in CDCl_3_ using dimethylacetamide (9.2 μL, 0.1 mmol) as internal standard. All β-nitroalcohol products are known and the spectroscopic data obtained from NMR analysis of the reaction mixtures were in agreement with those reported in the literature.

### 3.4. Typical Recycling Procedure

After reaction and extraction with EtOAc, the aqueous phase with remaining catalyst was freeze-dried prior addition of the reaction substrates and solvent for the next run.

### 3.5. Kinetic Studies

Reaction conversions were unequivocally calculated by ^1^H-NMR analysis of the reaction mixtures according to the integration of characteristic signals of the species in the reaction mixture in the presence of a suitable internal standard. Each experimental point represents the average of at least two experiments. Among various kinetics models, lines presented in the kinetic plots show best-fits of the first-order model for each case (i.e., (NO_2_R) ≥ (aldehyde)). Due to the fact that not all reactions reached 100% yield, data fitting was made according to the variation of ln((C_t_ − C_∞_ )/(C_∞_ − C_0_)) with time, where C_t_ is the concentration at a given time t; C_∞_ the final concentration (at infinite time) and C_0_ the initial concentration (at t = zero time). For reaction conversions close to 100%, plots of ln(C_t_/C_0_) versus time provided consistent results (C_∞_ = 0). Under these considerations, minor differences were observed between the exponential and linear fits. All errors reported for the rate constants *k* were calculated by graphical analysis. Kinetics data for other biopolymers have been previously reported by us and were used for comparison: Chitosan [18], gelatin [19], collagen [19], BSA [19], freeze-dried silk fibroin [20] and calcium alginate aerogel [21]. Sample preparation and details of the reactions can be found in the corresponding references.

## 4. Conclusions

In conclusion, we have demonstrated that keratin proteins are able to promote C-C bond formation via the nitroaldol (Henry) reaction between various aldehydes and nitroalkanes. Appropriate control experiments demonstrated the intrinsic catalytic activity of the keratin. Both aromatic and heteroaromatic aldehydes having strong or moderate electron-withdrawing groups were converted exclusively into the corresponding β-nitroalcohol products in both DMSO and in water in the presence of TBAB as phase transfer catalyst. In contrast, aromatic aldehydes bearing electron-donating groups and aliphatic aldehydes showed poor or no conversion, respectively. In general, the reactions in water/TBAB required twice the amount of reaction time than in DMSO to achieve similar conversions. Although the heterogeneous nature of the reaction allowed for the recovery and reuse of the keratin, a gradual deactivation of the catalyst was observed after each cycle. Comparative kinetic studies with other biopolymers revealed that the rate of the nitroaldol (Henry) reaction strongly depends on the nature of the biopolymer. The effect of different forms of keratins on the ability to catalyze this and other C-C bond forming reactions, as well as detailed mechanistic studies in both organic and aqueous medium, including different ionic liquids, are currently under study in our laboratories and the results will be reported in due course.

## Figures and Tables

**Table 1:**
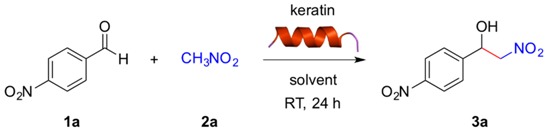
Initial solvent screening for the nitroaldol (Henry) reaction between **1a** and **2a** ^a^.

Entry	Solvent	Yield ^c^ of 3a (%)
1	Toluene	0
2	THF	0
3	CH_3_CN	0
4	CH_2_Cl_2_	11
5	CHCl_3_	10
6	DMSO	66 (0) ^d^
7	H_2_O	11
8	H_2_O/TBAB ^b^	57 (3) ^d^

^a^ Reaction conditions: **1a** (0.1 mmol), **2a** (1.0 mmol), keratin (10 mg), solvent (0.5 mL), RT, orbital shaking (150 rpm), 24 h; ^b^ TBAB (0.1 mmol). The addition of the phase transfer catalyst did not change the pH of the solution; ^c^ ^1^H-NMR yields of crude product obtained in the presence of dimethyl acetamide (DMA) as internal standard; ^d^ Background reaction without keratin.

**Table 2:**
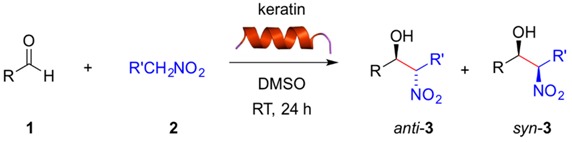
Keratin-catalyzed nitroaldol (Henry) reaction in DMSO ^a^.

Entry	R (1)	R′ (2)	Yield ^b^ of 3 (%)	3	dr ^d^ (*anti*/*syn*)
1	(4-NO_2_)-C_6_H_4_	H	84	3a	-
2	(4-NO_2_)-C_6_H_4_	CH_3_	96	3b	1:1
3	(3-NO_2_)-C_6_H_4_	H	93	3c	-
4	(3-NO_2_)-C_6_H_4_	CH_3_	98	3d	1:0.8
5	(2-NO_2_)-C_6_H_4_	H	94	3e	-
6	(2-NO_2_)-C_6_H_4_	CH_3_	96	3f	1:1.3
7	(4-F)-C_6_H_4_	H	10	3g	-
8	(4-Cl)-C_6_H_4_	H	25	3h	-
9	(3-Cl)-C_6_H_4_	H	48	3i	-
10	(4-Br)-C_6_H_4_	H	25	3j	-
11	(4-OH)-C_6_H_4_	H	5	3k	-
12	(4-CN)-C_6_H_4_	H	30	3l	-
13	(4-Me)-C_6_H_4_	H	7	3m	-
14	C_6_H_4_	H	9	3n	-
15	(4-CHO)-C_6_H_4_	H	65 ^c^	3o	-
16	Pyrid-2-yl	H	90	3p	-
17	Pyrid-2-yl	CH_3_	96	3q	1:1.7
18	Furfur-2-yl	H	3	3r	-
19	Naphtha-1-yl	H	13	3s	-

^a^ Reaction conditions: **1** (0.1 mmol), **2** (1.0 mmol), keratin (10 mg), DMSO (0.5 mL), RT, orbital shaking (150 rpm), 24 h; ^b^ Yield determined by ^1^H-NMR in the presence of DMA as internal standard. ^c^ Yield of monosubstituted β-nitroalcohol; ^d^ Diastereomeric ratio *anti*/*syn* determined by ^1^H-NMR analyses. “Not applicable” is represented by a dash (-).

**Table 3:**
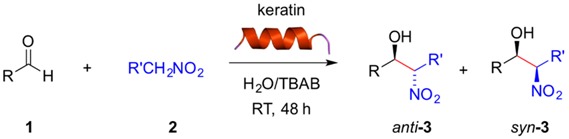
Keratin-catalyzed nitroaldol (Henry) reaction in H_2_O/tetrabutylammonium bromide (TBAB) ^a^.

Entry	R (1)	R’ (2)	Yield ^b^ of 3 (%)	3	dr ^e^ (*anti*/*syn*)
1	(4-NO_2_)-C_6_H_4_	H	73 (50) ^c^	3a	-
2	(4-NO_2_)-C_6_H_4_	CH_3_	94	3b	n.d.
3	(3-NO_2_)-C_6_H_4_	H	21	3c	-
5	(2-NO_2_)-C_6_H_4_	H	70	3e	-
6	(2-NO_2_)-C_6_H_4_	CH_3_	98	3f	1:0.8
7	(4-F)-C_6_H_4_	H	9	3g	-
8	(4-Cl)-C_6_H_4_	H	18	3h	-
9	(3-Cl)-C_6_H_4_	H	34	3i	-
10	(4-Br)-C_6_H_4_	H	14	3j	-
11	(4-OH)-C_6_H_4_	H	1	3k	-
12	(4-CN)-C_6_H_4_	H	66	3l	-
13	(4-CN)-C_6_H_4_	CH_3_	89	3m	n.d.
14	(4-Me)-C_6_H_4_	H	4	3n	-
15	C_6_H_4_	H	8	3o	-
16	(4-CHO)-C_6_H_4_	H	19 ^d^	3p	-
17	Pyrid-2-yl	H	72	3q	-
18	Pyrid-2-yl	CH_3_	85	3r	1:0.9
19	Furfur-2-yl	H	11	3s	-
20	Naphtha-1-yl	H	0	3t	-

^a^ Reaction conditions: **1** (0.1 mmol), **2** (1.0 mmol), TBAB (0.1 mmol), keratin (10 mg), H_2_O (0.5 mL), RT, orbital shaking (150 rpm), 48 h; ^b^ Yield determined by ^1^H-NMR in the presence of DMA as internal standard; ^c^ Yield of the reaction carried out in the presence of BMIM-PF_6_ (0.1 mmol) instead of TBAB. Reaction time = 72 h; ^d^ Yield of monosubstituted β-nitroalcohol; ^e^ Diastereomeric ratio *anti/syn* determined by ^1^H-NMR analyses. Abbreviation: n.d. = not determined. “Not applicable” is represented by a dash (-).
